# Iron Deficiency Anemia Coexists with Cancer Related Anemia and Adversely Impacts Quality of Life

**DOI:** 10.1371/journal.pone.0163817

**Published:** 2016-09-28

**Authors:** Giridhar Kanuri, Ritica Sawhney, Jeeva Varghese, Madonna Britto, Arun Shet

**Affiliations:** 1 Wellcome Trust- DBT Hematology Research Unit, St. Johns Research Institute, Bangalore, Karnataka, India; 2 College of Nursing, St. John’s National Academy of Health Sciences, Bangalore, Karnataka, India; 3 Department of Medical Oncology, St. Johns Medical College Hospital, Bangalore, Karnataka, India; Pennsylvania State University College of Medicine, UNITED STATES

## Abstract

Cancer related anemia (CRA) adversely affects patient Quality of Life (QoL) and overall survival. We prospectively studied the prevalence, etiology and the impact of anemia on QoL in 218 Indian cancer patients attending a tertiary referral hospital. The study used the sTfR/log Ferritin index to detect iron deficiency anemia and assessed patient QoL using the Functional Assessment of Cancer Therapy-Anemia (FACT-An) tool, standardized for language. Mean patient age was 51±13 years and 60% were female. The prevalence of cancer related anemia in this setting was 64% (n = 139). As expected, plasma ferritin did not differ significantly between anemic (n = 121) and non-anemic cancer patients (n = 73). In contrast, plasma sTfR levels were significantly higher in anemic cancer patients compared to non-anemic cancer patients (31 nmol/L vs. 24 nmol/L, p = 0.002). Among anemic cancer patients, using the sTfR/log Ferritin index, we found that 60% (n = 83) had iron deficiency anemia (IDA). Interestingly, plasma sTfR levels were significantly higher in cancer patients with CRA+IDA (n = 83) compared with patients having CRA (n = 38) alone (39 nmol/L vs. 20 nmol/L, p<0.001). There was a significant linear correlation between Hb and QoL (Spearman ρ = 0.21; p = 0.001) and multivariate regression analysis revealed that every gram rise in Hb was accompanied by a 3.1 unit increase in the QoL score (95% CI = 0.19–5.33; p = 0.003). The high prevalence of anemia in cancer patients, a major portion of which is due to iron deficiency anemia, the availability of sensitive and specific biomarkers of iron status to detect IDA superimposed on anemia of inflammation, suggests an urgent need to diagnose and treat such patients. Despite the potential negative consequences of increasing metabolically available plasma iron in cancer, our clinical data suggest that detecting and treating IDA in anemic cancer patients will have important consequences to their QoL and overall survival. Clinical trials of iron therapy in these patients will be able to demonstrate the potential for benefit or harm.

## Introduction

The incidence of anemia is high among cancer patients with 39% anemic at the time of diagnosis of the cancer [1, 2] and a further 13% of non anemic cancer patients developing anemia during the course of their treatment/disease [3]. The incidence of cancer related anemia (CRA) varies depending on the type of malignancy, stage, duration of the disease, intensity and type of tumor therapy regimen, and the occurrence of intercurrent infections or surgery [4, 5]. CRA confers an overall adverse prognosis particularly in anemic patients with lung, prostate, and head and neck cancer as well as lymphoma, which have a significant reduction in survival compared with their non-anemic counterparts [6]. Furthermore, anemia *per se* results in cardiorespiratory compromise leading to symptoms associated with a reduced quality of life (QoL) [1].

There is a growing body of evidence that both functional status and QoL are significantly compromised in cancer patients with hemoglobin (Hb) values ≤12 g/dL [6–8]. Moderately anemic cancer patients (Hb, 8–10 g/dL) exhibit fatigue, lethargy, dyspnea, loss of appetite and inability to concentrate, affecting their overall QoL [9]. A major etiological factor for cancer-related anemia is iron deficiency, particularly in low middle income settings [10]. Even in the absence of anemia, iron deficiency is correlated with impaired physical function and fatigue [11]. However, very few studies in the Asian context report the prevalence and etiology of anemia in cancer patients. Since retrospective studies in our setting demonstrating a high prevalence of CRA [12] and population survey data revealed high iron deficiency anemia prevalence, we hypothesized that anemia in cancer patients would be predominantly due to iron deficiency. To test this hypothesis, we undertook a cross-sectional study of cancer patients undergoing treatment at a tertiary medical centre in South India and assessed the prevalence and etiology of anemia and its relationship to QoL.

## Materials and Methods

### Subjects

**Fig 1:**
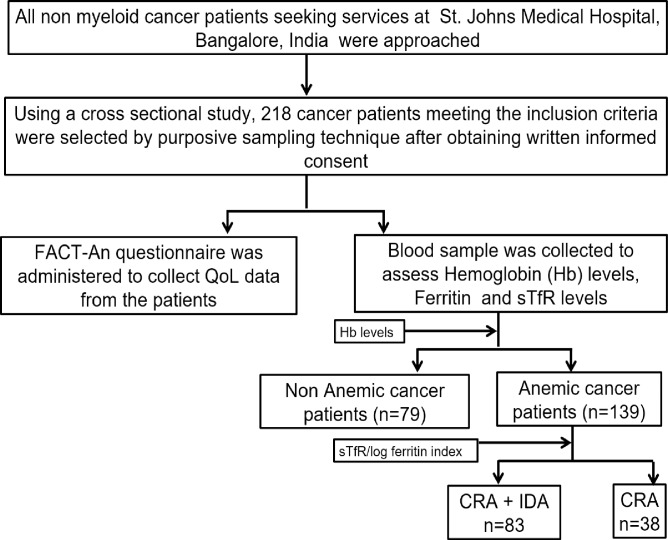
Schematic representation of the study design Using gender adjusted WHO norms [13] patients were divided into anemic and non anemic categories. Anemic cancer patients were further subdivided into those having only cancer related anemia (CRA) or those having cancer related anemia patients combined with iron deficiency anemia (CRA+IDA).

Using a cross-sectional study design, 218 cancer patients attending a tertiary medical center oncology clinic in 2013–2014 were prospectively enrolled (Fig 1). We excluded the following patients either due to practical reasons or to minimize confounding: (i) patients who were in the immediate post-operative period or/and critically ill, (ii) patients diagnosed with myeloid malignancies, (iii) patients unaware of the diagnosis of cancer, (iv) patients who were recently transfused with erythrocytes in the 3 months prior to inclusion. The characteristics of patients are detailed in Table 1. The study protocol was approved by the Institutional Ethical Committee of St. Johns Medical College and Hospital (Bangalore, India). Written informed consent was obtained from all participants and the study was conducted according to the principles expressed in the Declaration of Helsinki.

**Table 1:** Characteristics of cancer patients.

	Cancer patients
*N*	218
Sex, % female	60
Age, Y^1^	51+13
**Type of cancer (%) and Hb levels (g/dL)**	
Breast %	37
Hb levels^1^	11.3 + 1.6
Gastro intestinal %	17
Hb levels^1^	11.1 + 2.0
Lymphoma %	15
Hb levels^1^	10.5 + 2.6
Head and Neck %	10
Hb levels^1^	12.7 + 2.1
Lung %	7
Hb levels^1^	10.9 + 2.5
**Stage of cancer patients (%) and Hb levels (g/dL)**	
Stage I %	7.7
Hb levels^1^	11.3 + 1.8
Stage II %	19
Hb levels^1^	11.4 + 2.0
Stage III %	24
Hb levels^1^	11.1 + 2.0
Stage IV %	26
Hb levels^1^	11.3 + 1.8
**Hb levels (g/dL) in CRA and CRA + IDA patients**	
CRA patients (n = 38)	10.3 + 1.4
CRA + IDA patients (n = 83)	9.9 + 1.8
**Treatment Regimen**	
No Treatment	59
Chemotherapy	60
Surgery	36
Radiation	1
Chemo+Surgery	30
Chemo+Radiation	10
Chemo+Radiation+Surgery	22

^1^ Values are means of ± SD. CRA; cancer related anemia, CRA+IDA; cancer related anemia + iron deficiency anemia

### Samples

Blood samples collected by venous phlebotomy into EDTA containing tubes were centrifuged at 1200*g* to obtain plasma and stored at -80°C until future analysis. Complete blood counts were performed within 6 hours of phlebotomy using an automated cell counter (Sysmex Xs-800i, Sysmex, Japan). Peripheral blood smears were performed on all samples exhibiting macrocytosis and evaluated for megaloblastic changes to rule out the presence of B12 and Folate deficiency.

### Functional Assessment of Cancer Therapy-Anemia (FACT-An) questionnaire

QoL was assessed using the FACT-An questionnaire. The total score is calculated as the sum of the individual scores on questions related to physical wellbeing, social and family wellbeing, emotional wellbeing and functional wellbeing, in addition to 13 fatigue and 7 anemia-specific questions. We previously tested and validated this tool in three regional languages (Kannada, Tamil and Telugu) for language and contextual relevance.

### Ferritin, soluble transferrin receptor (sTfR) and the index

Plasma ferritin and sTfR levels were quantitatively determined by paramagnetic particle chemiluminescent immunoassay method using the Access 2 Immunoanalyzer (Access 2, Beckmann Coulter Inc). The immunoanalyzer allows simultaneous analysis of both analytes on the same sample specimen.

### Definition of iron deficiency anemia (IDA) superimposed on cancer related anemia (CRA)

For this study, we used the following definitions:

1. Anemia was defined according to age and sex appropriate WHO criteria [13]. In this study, males with Hb values <13g/dL and females with < 12 g/dL were considered to have anemia.
2. CRA was defined as the presence of anemia with a sTfR/log ferritin value of <1.03 based on previously published data [14].
3. IDA superimposed on CRA was diagnosed as a sTfR/log ferritin value of ≥1.03. Ferritin index was calculated by dividing the absolute value of sTfR with log ferritin value [14].

### Statistical analysis

Variables with a Gaussian distribution are presented as mean ± SD, whereas for variables distributed in a non-Gaussian manner, the data are shown as medians with interquartile ranges. Univariate analysis between hemoglobin values and FACT-An scores were performed using the Spearman test. Multivariate analysis using regression modeling was done to evaluate QoL and Hb levels to determine effect of confounding by other factors such as age, stage of cancer and chemotherapy. Statistical differences between groups under comparison were determined using the Mann-Whitney U test. P value < 0.05 was considered to be significant. All statistical analyses were done with SPSS 16.0 software (SPSS Inc., Chicago, IL).

## Results

### Patient characteristics

Out of 236 patients, 218 patients fulfilled the inclusion criteria and were enrolled into the study. Their mean age was 51±13 years, and there were 131 female (60%) and 87 male patients. Among anemic cancer patients (n = 139), 26% (n = 37) had anemia at the time of enrollment and the remaining 74% (n = 102) had undergone some form of treatment (Chemotherapy/Surgery/Radiation). Breast cancer (37%) was the most frequent tumor type followed by gastrointestinal (17%), lymphoma (15%) and head and neck cancers (10%). The majority of patients had stage IV disease (26%) followed by stage III (24%) (Table 1).

### Hematological parameters

A large proportion of the study population (64%) was anemic. Mean Hb in cancer patients without anemia was 13.3 ± 1 g/dL and in cancer patients with anemia was 10.2 ± 1.5 g/dL (p <0.001). When patients were categorized according to the type of cancer, the mean Hb value was lowest in patients with a diagnosis of lymphoma (10.5 ± 2.6 g/dL) followed by lung cancer (10.9 ± 2.5 g/dL). There were no demonstrable differences in mean Hb values based on patients cancer stage (Table 1).

### Biochemical parameters to establish anemia etiology

**Fig 2:**
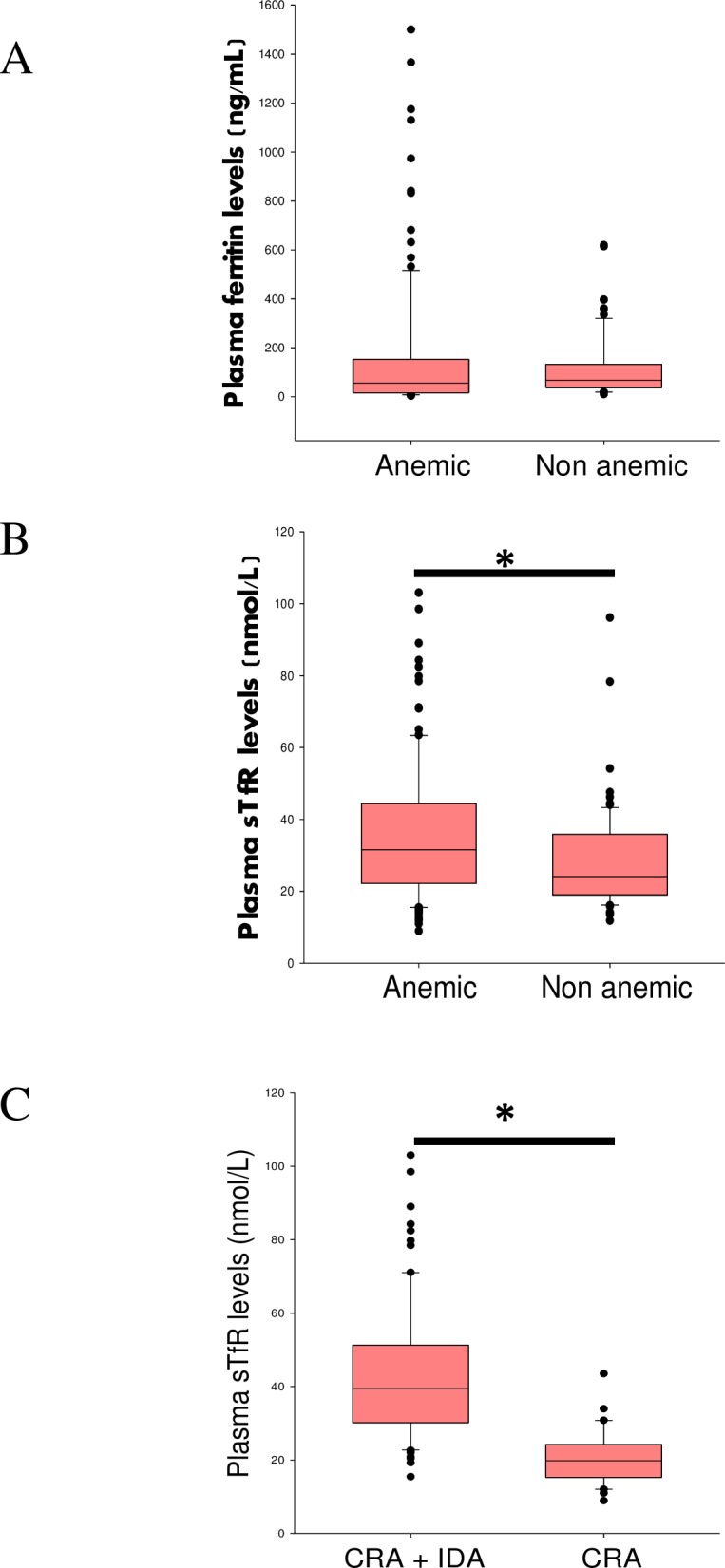
Ferritin, sTfR levels in cancer patients. (A & B) plasma ferritin and sTfR levels in anemic (n = 121) and non anemic cancer patients (n = 73), * p< 0.05 compared with anemic group. (C) plasma sTfR levels in CRA (n = 38) and combined CRA+IDA patients (n = 83), * p< 0.05 compared with CRA patients.

Plasma ferritin values were above the normal reference range due to the presence of inflammation associated with cancer. There were no observable differences in ferritin levels between anemic and non-anemic cancer patients (55 ng/ml vs. 66 ng/ml, p = 0.1) (Fig 2A). Levels of plasma sTfR were significantly higher in cancer patients with anemia compared to cancer patients without anemia (31 nmol/L vs. 24 nmol/L, p = 0.002) (Fig 2B). Using the sTfR/ferritin index as a biochemical means of detecting iron-deficiency anemia combined with CRA, we found that over 60% of cancer patients had both CRA and IDA. Interestingly, the plasma sTfR levels in CRA+IDA (39 nmol/L vs. 20 nmol/L, p<0.001) patients were significantly higher compared with levels in patients having only CRA (Fig 2C).

### Assessment of Quality of Life scores

**Fig 3:**
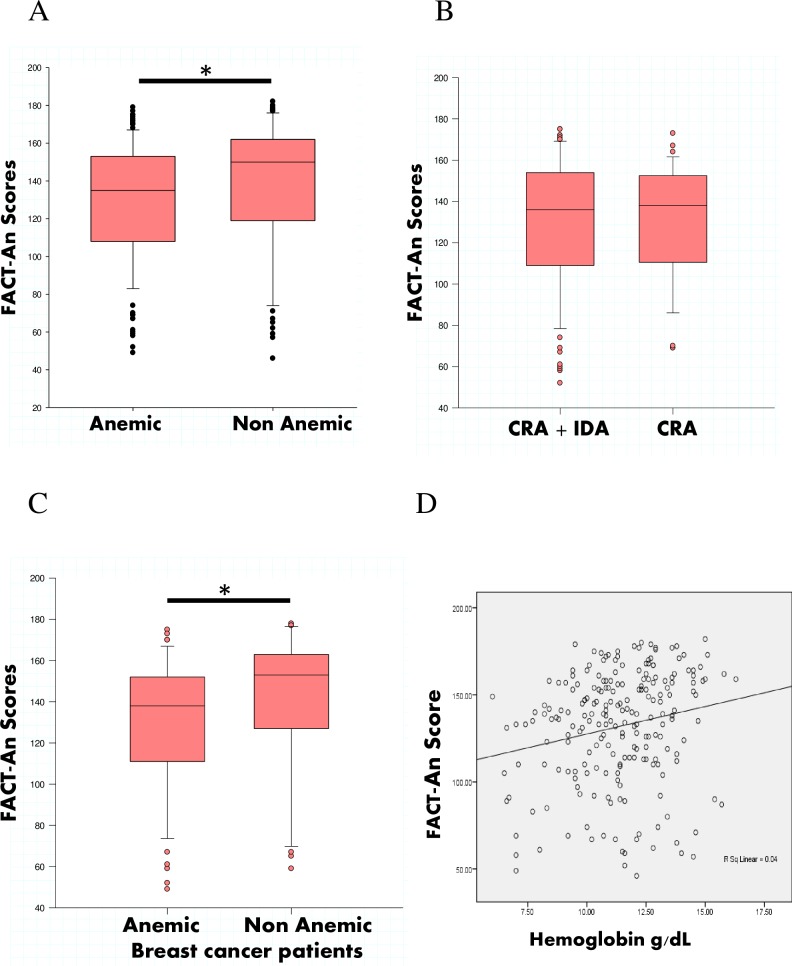
Correlation analysis as well as FACT An scores in anemic and non-anemic cancer patients. (A) FACT-An scores were compared between anemic (n = 139) and non anemic cancer patients (n = 79), * p< 0.05 compared with anemic group. (B) FACT-An scores was compared between CRA+ IDA (n = 83) patients and only CRA (n = 38) patients. (C) FACT-An Scores were compared between anemic (n = 52) and non anemic breast cancer patients (n = 33), * p< 0.05 compared with anemic breast cancer patients. (D) Spearman correlation was used to correlate Hb levels and FACT-An scores. Correlation coefficient was ρ = 0.21; p = 0.001.

Studying the functional and anemia status of cancer patients, we found that QoL as assessed by the Fact-An tool was significantly higher in non-anemic cancer patients than anemic cancer patients (Fact-An Score: 150 units vs. 135 units, p = 0.01) (Fig 3A). After stratification of cancer patients into groups with CRA alone and those with combined CRA+IDA, we found no differences in QoL between CRA patients and CRA+IDA patients (FACT-An Score: 138 units vs. 136 units p = 0.9) (Fig 3B).

When categorized according to type of cancer, only breast cancer patients without anemia were found to have significantly higher quality of life in comparison to their anemic counterparts (FACT-An Score: 153 units vs. 138 units, p = 0.02) (Fig 3C). There was a significant correlation between Hb levels and FACT-An scores in all cancer patients, demonstrating that higher Hb levels were associated with a better QoL (Fig 3D). Using multivariate regression analysis, there was an increase of 3.1 units on the FACT-An score for every gram rise in Hb level (p = 0.003; CI = 0.19–5.33).

## Discussion

The results of this study demonstrate 3 key observations: (1) the prevalence of anemia is high in cancer patients (64%), consistent with findings from other studies [1, 15–19] (2) the etiology of cancer related anemia in this setting is mostly iron deficiency anemia, and (3) there is a significant association between hemoglobin level and quality of life in cancer patients.

The anemia prevalence of 64% seen in our study is slightly higher compared to findings from other studies from European and Asia Pacific region. The ECAS study, which was the first to provide information on prevalence of anemia in cancer patients, found 39% of prevalence at enrollment [1]. A recent cross sectional study of 214 non-myleoid tumor patients in 21 Spanish hospitals found that the prevalence of anemia was 48% [15], while a study conducted in Italy and Austria with 1136 solid tumor patients showed an overall anemia prevalence of 31% [16]. However, a study of solid tumor patients presenting at Belgian oncology and hematology centers found a lower prevalence of 13.8% [17]. The Australian Cancer Anemia Survey conducted during the same time as the ECAS showed a 35% anemia prevalence in cancer patients at the time of enrollment [18]. A single centre retrospective study in 148 patients with solid tumors in Japan reported that 44% had anemia at enrollment [19]. Our finding of a higher anemia prevalence among cancer patients compared with these studies is probably due in part to a higher due to prevaling malnutrition in general population [20, 21] and possibly due to a higher number of female cancer patients in the study. Review of the clinical history of the population with CRA in our study revealed 26% had anemia prior to initiating antineoplastic treatment but the remaining 74% had received some form of treatment prior to study enrolment (e.g. chemotherapy, radiation, surgery or a combination of these treatments). Thus, a mixed population of treated and untreated patients in this study could also have contributed to a higher anemia prevalence in our study.

The etiology of CRA is multifactoral, but the major contributor appears to be iron restricted erythropoiesis resulting from anemia of inflammation, absolute iron deficiency or a combination of the two [22, 23]. The sTfR/log ferritin index can identify iron deficiency anemia superimposed on anemia of inflammation at a cut off value of 1.03 [14, 24–26]. Using this index, we found that the major etiology of anemia in these cancer patients was iron deficiency anemia superimposed on anemia of inflammation [22]. Our findings of significantly elevated sTfr levels in anemic cancer patients with IDA compared to healthy controls (data not shown), suggest that cancer *per se* neither decreased erythropoietic activity nor suppressed iron mobilization [27] and are in keeping with the findings of Zubirkhina et al [28]. These data indicate that preexsisting micronutritient deficiencies (e.g. iron deficiency) profoundly influence the development of cancer related anemia, in addition to chemotherapy and tumor cell-released cytokines.

Results of Lind et al [29] suggest that the QoL in cancer patients is likely to be improved with increasing Hb levels. Indeed, Crawford et al [30] showed an improved QoL with a 1 gm increase in hemoglobin from 11g/dL to 12g/dL. More recently, a randomized controlled study of a combination of erythropoiesis stimulating agents (ESA) and intravenous iron resulted in improved hemoglobin levels (Baseline Hb: 9.7±0.8 g/dL, End of the study: +Δ 2.5g/dL) and significantly improved QoL (p = 0.0001) [31]. In our study, maximal incremental gain in QoL occurred when hemoglobin was >13g/dL (Table 2).

**Table 2:** Distribution of hemoglobin levels and FACT-An scores in cancer patients.

Hemoglobin groups	6–9 g/dL	>9–11 g/dL	>11–13 g/dL	>13g/dL
*n*	28 (13%)	66 (30%)	79 (36%)	45 (21%)
FACT-An Score^1^	132 (82–139)	139 (115–154)	139 (113–160)	150 (117–161)*

^1^ Data represented as median, (inter quartile range)

* P = 0.01 compared with 6-9g/dL hemoglobin group

Although QoL was lower in cancer patients with iron deficiency anemia and iron restricted erythropoiesis compared with cancer patients with iron restricted erythropoiesis alone, these differences did not reach the level of significance (p = 0.9) possibly due to the variability of FACT-An scores and the small sample size of this subgroup. The significant positive correlation observed between and Hb and QoL in our study, is consistent with the findings of Wasada I et al [32], where improvements in Hb in cancer patients over a three month period positively correlated with changes in QoL scores.

One of the major strengths of the study is the inclusion of a broad range of cancers and careful detection of iron deficiency anemia in a population with a high pre test probability of having a micronutrient deficiency. However, the results of this study have to be interpreted cautiously since the study recruited subjects from a single center and did not evaluate the effect of therapeutic iron on hemoglobin or patient outcomes. Nevertheless, this is the first study to rigorously determine the prevalence and etiology of cancer-related anemia in India and strengthens the notion that increments in Hb level would improve the QoL of cancer patients with anemia.

## Conclusion

This study has shown for the first time that the major etiology of anemia in Indian cancer patients is iron deficiency anemia. sTfR/log ferritin index may be used as a marker in differential diagnosis of iron restricted erythropoiesis vs. absolute iron deficiency in cancer patients. Additional studies evaluating the impact of therapeutic iron in IDA in patients with CRA [33] and QoL are warranted, as are studies examining the negative consequences of increasing metabolically available iron in cancer patients [34].
